# *Dirofilaria* spp. and *Angiostrongylus vasorum*: Current Risk of Spreading in Central and Northern Europe

**DOI:** 10.3390/pathogens10101268

**Published:** 2021-10-01

**Authors:** Hans-Peter Fuehrer, Simone Morelli, Maria Sophia Unterköfler, Anna Bajer, Karin Bakran-Lebl, Dorota Dwużnik-Szarek, Róbert Farkas, Giulio Grandi, Mike Heddergott, Pikka Jokelainen, Tanja Knific, Michael Leschnik, Martina Miterpáková, David Modrý, Heidi Huus Petersen, Karl Skírnisson, Aleksandra Vergles Rataj, Manuela Schnyder, Christina Strube

**Affiliations:** 1 Department of Pathobiology, Institute of Parasitology, University of Veterinary Medicine Vienna, Veterinärplatz 1, 1210 Vienna, Austria; Maria.Unterkoefler@vetmeduni.ac.at (M.S.U.); karin.bakran-lebl@vetmeduni.ac.at (K.B.-L.); 2 Faculty of Veterinary Medicine, University of Teramo, 64100 Teramo, Italy; smorelli@unite.it; 3 Department of Eco-Epidemiology of Parasitic Diseases, Institute of Developmental Biology and Biomedical Sciences, Faculty of Biology, University of Warsaw, Miecznikowa 1, 02-096 Warsaw, Poland; anabena@biol.uw.edu.pl (A.B.); dorota.dwuznik@biol.uw.edu.pl (D.D.-S.); 4 Department of Parasitology and Zoology, University of Veterinary Medicine, 1078 Budapest, Hungary; Farkas.Robert@univet.hu; 5 Section for Parasitology, Department of Biomedical Sciences and Veterinary Public Health, Swedish University of Agricultural Sciences (SLU), 750 07 Uppsala, Sweden; giulio.grandi@slu.se; 6 Department of Microbiology, National Veterinary Institute (SVA), 756 51 Uppsala, Sweden; 7 Department of Zoology, Musée National d’Historire Naturelle, 25, Rue Münster, 2160 Luxembourg, Luxembourg; mike-heddergott@web.de; 8 Department of Bacteria, Parasites and Fungi, Infectious Disease Preparedness, Statens Serum Institut, Artillerivej 5, DK-2300 Copenhagen S, Denmark; PIJO@ssi.dk; 9 Institute of Food Safety, Feed and Environment, Veterinary Faculty, University of Ljubljana, Gerbičeva 60, 1000 Ljubljana, Slovenia; tanja.knific@vf.uni-lj.si; 10 Clinical Unit of Internal Medicine Small Animals, Department/Universitätsklinik für Kleintiere und Pferde, University of Veterinary Medicine Vienna, Veterinärplatz 1, 1210 Vienna, Austria; Michael.leschnik@vetmeduni.ac.at; 11 Institute of Parasitology, Slovak Academy of Sciences, Hlinkova 3, 040 01 Košice, Slovakia; miterpak@saske.sk; 12 Biology Center, Institute of Parasitology, Czech Academy of Sciences, 37005 Ceske Budejovice, Czech Republic; ModryD@VFU.cz; 13 Department of Veterinary Sciences, Faculty of Agrobiology, Food and Natural Resources/CINeZ, Czech University of Life Sciences Prague, 16500 Praha-Suchdol, Czech Republic; 14 Department of Botany and Zoology, Faculty of Science, Masaryk University, 61137 Brno, Czech Republic; 15 Centre for Diagnostic, Department of Health Technology, Technical University of Denmark, 2800 Kongens Lyngby, Denmark; hhpet@dtu.dk; 16 Institute for Experimental Pathology at Keldur, University of Iceland, Keldnavegur 3, 112 Reykjavik, Iceland; karlsk@hi.is; 17 Institute of Microbiology and Parasitology, Veterinary Faculty, University of Ljubljana, Gerbičeva 60, 1000 Ljubljana, Slovenia; Aleksandra.VerglesRataj@vf.uni-lj.si; 18 Institute of Parasitology, Vetsuisse Faculty, University of Zurich, 8057 Zürich, Switzerland; Manuela.Schnyder@uzh.ch; 19 Centre for Infection Medicine, Institute for Parasitology, University of Veterinary Medicine Hannover, 30559 Hannover, Germany; Christina.Strube@tiho-hannover.de

**Keywords:** Central Europe, Northern Europe, *Dirofilaria immitis*, *Dirofilaria repens*, *Angiostrongylus vasorum*

## Abstract

In the past few decades, the relevance of *Dirofilaria immitis* and *Dirofilaria repens*, causing cardiopulmonary and subcutaneous dirofilariosis in dogs and cats, and of *Angiostrongylus vasorum*, causing canine angiostrongylosis, has steadily increased in Central and Northern Europe. In this review, a summary of published articles and additional reports dealing with imported or autochthonous cases of these parasites is provided for Central (Austria, Czechia, Germany, Hungary, Luxemburg, Poland, Slovakia, Slovenia, and Switzerland) and Northern (Denmark, Finland, Iceland, Norway, and Sweden) Europe. Research efforts focusing on *Dirofilaria* spp. and *A. vasorum* have varied by country, and cross-border studies are few. The housing conditions of dogs, pet movements, the spread of competent vectors, and climate change are important factors in the spread of these nematodes. Dogs kept outside overnight are a major factor for the establishment of *Dirofilaria* spp. However, the establishment of invasive, diurnal, synanthropic, competent mosquito vectors such as *Aedes albopictus* may also influence the establishment of *Dirofilaria* spp. The drivers of the spread of *A. vasorum* remain not fully understood, but it seems to be influenced by habitats shared with wild canids, dog relocation, and possibly climatic changes; its pattern of spreading appears to be similar in different countries. Both *Dirofilaria* spp. and *A. vasorum* merit further monitoring and research focus in Europe.

## 1. Introduction

In the past few decades, arthropod-borne and gastropod-borne pet diseases have changed their distribution. A series of drivers, including wildlife-habitat reduction, urbanization, climatic changes, increased movements of pets traveling with their owners, and animal rehoming, have favored the geographical spread of specific arthropod-borne and gastropod-borne diseases within endemic areas, and their emergence in previously free areas [1,2,3,4,5,6,7]. In Europe, this has led to the modification of the epizootiological picture of diseases with key relevance in veterinary medicine, e.g., of cardiopulmonary and subcutaneous dirofilariosis caused by *Dirofilaria immitis* and *Dirofilaria repens*, respectively, and of canine angiostrongylosis due to *Angiostrongylus vasorum*, which have expanded their geographical distribution [4,8,9,10,11,12,13].

Canines are the main definitive hosts and primary reservoir of *D. immitis* and *D. repens* [9,14,15]. Nevertheless, these filarioids have moderate host specificity and are able to infect a wide range of vertebrates, also including cats and humans [9,14,15,16,17,18,19].

Adults of *D. immitis* live in the pulmonary arteries and, occasionally, the right chambers of the hearts of definitive hosts. After mating, females release first-stage larvae (L1), known as microfilariae, into the bloodstream. During the blood meal, microfilariae are picked up by mosquitoes, within which they develop to the third infective larval stages (L3). When feeding on vertebrate hosts, infected mosquitoes transmit L3, which undergo two further larval stages and then reach the adult stage with patency of six months [14,15]. Canine heartworm disease (HWD), caused by *D. immitis*, is usually a chronic cardiorespiratory disease that can be fatal if not treated [20]. Clinical signs include cough, exercise intolerance, dyspnea, and ascites; in severe cases, pulmonary hypertension, heartworm thromboembolism, and heart failure may occur [15,20]. Cats are less suitable hosts than dogs; they usually harbor a low number of adult *D. immitis*, and patent infections are rare [17]. In cats, the arrival and early death of immature adults in the pulmonary arteries causes a marked inflammatory response known as heartworm-associated respiratory disease (HARD), characterized by dyspnea, cough, anorexia, and vomiting [17,21]. Cats that survive the HARD phase can become subclinically infected until the death of adult heartworms, which may result in a sudden fatal outcome [17]. Human infections with *D. immitis* usually result in pulmonary granulomas known as “coin lesions”, and can be asymptomatic or present with cough and nonspecific signs [14,22]. The available literature has no reports of human infections with microfilaremia.

The lifecycle of *D. repens* is very similar to the one of *D. immitis*, with the main difference represented by the final localization of the adult stages in the vertebrate hosts, i.e., the subcutaneous and intramuscular connective tissues [9]. Most canine infections are subclinical; in the case of the appearance of clinical signs, non-painful subcutaneous nodules, pruritus, erythema, alopecia, and papulae can be observed [23,24,25]. The disease in cats is similar, with pruritus, alopecia, erythema, and papulae as the most frequent clinical manifestations [24]. *Dirofilaria repens* has higher zoonotic potential if compared to *D. immitis*, and thereby has public-health relevance in Europe [9]. Human infection with *D. repens* can present as subcutaneous nodules, mostly in the facial region, perioral and periorbital tissue, scrotum and testicles in men, and breasts in women; it is rarely microfilaremic [9,26].

*Dirofilaria immitis* and *D. repens* are traditionally endemic in Southern and Eastern Europe [9,10,27]. Nevertheless, a recent increase in the number of cases was reported in Central and Northern Europe, and adjacent regions for both nematodes [9,10,11,12,13,18]. Global warming is regarded as a key factor involved in the spreading of *Dirofilaria* spp., as it enhances the development of these filarioids inside mosquitoes [9,28].

Canine angiostrongylosis has been a predominant disease in canine veterinary medicine in the last 10 years. Similar to *D. immitis*, adults of *A. vasorum* inhabit the pulmonary arteries of definitive hosts [8]. After mating, females lay eggs that hatch and release L1, penetrate the alveoli, reach the pharynx, and then are swallowed and excreted with feces into the environment [8,29,30]. Thereafter, L1 penetrate or are ingested by a terrestrial gastropod (e.g., snails or slugs), within which they develop to L3. Dogs become infected when ingesting infected molluscs [29,31]. The diagnosis of canine angiostrongylosis is challenging for veterinary practitioners. Clinical pictures may be extremely variable, as (i) they range from subclinical to hyperacute, and (ii) infected dogs can display a plethora of different clinical signs that are both cardiorespiratory and nonspecific, gastrointestinal, neurological, and related to coagulation disorders [32,33,34,35,36]. The definitive host spectrum of *A. vasorum* is narrower than that of *Dirofilaria* spp., as it infects almost exclusively canids, with only few descriptions in other animals [8,37,38,39,40]. A single case of a natural non-patent infection was documented in a domestic cat [41], though the importance of feline angiostrongylosis due to *A. vasorum* or *Angiostrongylus chabaudi* is considered to be minimal at present, with patent infections reported only in wildcats [42,43]. At present, *A. vasorum* does not have any relevance to human health.

Several new records in both definitive and intermediate hosts documented a geographical expansion of *A. vasorum* in previously free areas, including the Iberian Peninsula [44,45] and Eastern [46,47,48,49,50], Central, and Northern Europe [51,52,53,54].

**Figure 1:**
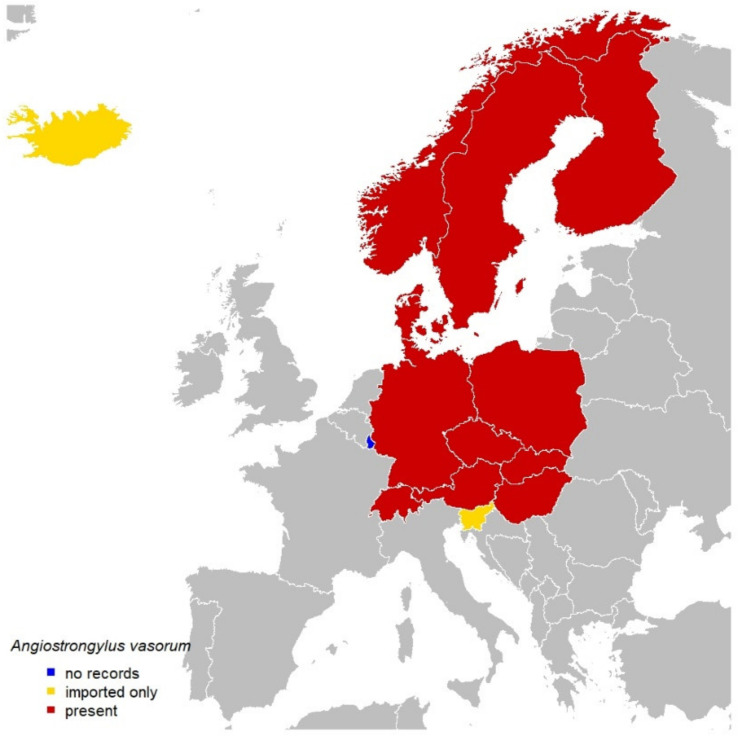
Distribution of *Angiostrongylus vasorum* in Central and Northern Europe.

The geographical expansion of *Dirofilaria* spp. and *A. vasorum* in areas that were previously considered to be non-enzootic requires constant epizootiological surveillance (Figure 1). These changes could partly be attributed to the increased interest of scientists and the pharma industry (funding studies), also strongly contributing to the development of new treatment options. An increase in the awareness of local veterinarians and owners is warranted, as there is a lack of updated data in many countries, and dogs may be at risk of infection with both *Dirofilaria* spp. and *A. vasorum*, even in areas where their presence is currently unexpected. The situation is a challenge for the veterinary profession, and regarding the zoonotic *Dirofilaria* spp., also for medical profession, particularly in areas where the parasites have recently emerged or are not yet established [55,56].

Although the number of studies has increased in the last few years, epizootiological knowledge on *Dirofilaria* spp. and *A. vasorum* in Central and Northern Europe is still fragmentary. Therefore, the aim of the present study is to comprehensively review epizootiological data from countries of Central and Northern Europe in order to provide an updated and accurate picture on canine dirofilariosis and angiostrongylosis.

## 2. *Dirofilaria* spp.

### 2.1. Central Europe

#### 2.1.1. Austria

The first diagnosed cases of imported canine *D. immitis* infection in Austria were published in 1987 and 1988 [57,58]. The first documented canine *D. repens* infection and mixed dirofilarial infection in Austria, all of them imported cases, were published in 2001 [59]. Between 2000 and 2007, another six *D. immitis* and four *D. repens* infections in imported dogs were documented [60]. In a local survey in Eastern Austria in 2008, seven out of 98 canine blood samples tested positive for *D. repens* by PCR—two dogs were not reported to have had any stay abroad; this may have documented the first autochthonous infections [61]. Seven years later, a review on canine and human dirofilarial cases in Austria reported 37 dogs with *D. repens* infection (including the seven possibly autochthonous infections) and 25 dogs with *D. immitis* infection till 2014—a total of 62 cases within 18 years [62]. In the four following years, 84 more cases were documented till 2018, mostly *D. immitis* infections from imported dogs, and 10 additional dogs with coinfections of *D. repens* and *D. immitis* [63]. The most recent surveys in dogs and mosquitos in Austria focused on the possible risk for the development of new local endemic foci in and near Austrian dog shelters, and possible infections in kenneled military dogs. In total, 115 shelter dogs from 14 animal shelters located in five different Austrian states were examined in 2018 and 2019. Blood samples were screened for *D. immitis*, using rapid diagnostic devices (SNAP 4Dx Plus, IDEXX Laboratories, Inc., Westbrook, ME, USA), PCR, and microscopical examination for microfilariae. In total, 91.0% of the dogs originated from countries endemic for dirofilariosis. Eleven dogs (9.6%), all originating from Hungary, tested positive for *D. immitis*. All mosquitos (n = 205) trapped in animal-shelter proximity tested negative for *Dirofilaria* spp. by PCR. Of these mosquitos, 98.5% belonged to a species proven or even suspected to transmit *Dirofilaria* spp. [64]. In the Military Working Dog Training Centre in Eastern Austria, two of 96 dogs tested positive for *D. repens* – one from Hungary and one originating from Austria [65]. Moreover, the first autochthonous case of *D. immitis* was recently documented in a cat from Burgenland [66]. Neither *D. repens* nor *D. immitis* have yet been reported from wild canids in Austria (e.g., red foxes (*Vulpes vulpes*)) [67].

The first reported human case of subcutaneous dirofilariasis in an Austrian woman was published in 1981. It was assumed that this infection had been imported from the Mediterranean area [68]. In 2006, the first autochthonous infection with *D. repens* in an Austrian woman near the Hungarian border was diagnosed [69]. From 1978 to 2014, 33 cases of human dirofilariasis caused by *D. repens* were reported in Austria (30 cases), and three cases caused by *D. immitis*, rising to a total of 39 cases in 2020. Over the past four decades, incidence has markedly increased, particularly after 1998 [62,70,71]. In 2018, Austria was classified as endemic for *D. repens* (but not for *D. immitis*) with sporadic human cases [9].

*D. repens* in mosquitos in Eastern Austria was first detected by PCR in 2012. A low local prevalence was supposed, as two of 437 pools of collected mosquitoes close to the Hungarian border gave a positive result. All 18 individuals of one positive pool belonged to the *Anopheles maculipennis* (Meigen, 1880) group, and 14 individuals in the other positive pool were the *Anopheles algeriensis* (Theobald, 1903) species [72]. In 2013–2015, 45,848 mosquitos were sampled and analyzed for filarioid DNA by PCR. The DNA of *D. repens* was found in an *Anopheles plumbeus* mosquito close to the Slovakian border, confirming that *D. repens* is still endemic in low prevalence in Eastern Austria [73]. Potential invasive mosquitoes are competent vectors of *D. repens* and *D. immitis*. *Aedes japonicus* eggs were identified in Lower Austria, Styria, and Burgenland. *Ae. japonicus* was first found in Vienna in July 2017 during a routine sampling of adult mosquitoes [74]. A survey in Western Austria in 2018 found *Ae. albopictus* and *Ae. japonicus* eggs at highways and urban areas in both East and North Tyrol [75]. *Ae. albopictus* was first recorded in Vienna, Austria in August 2020. The species occurred in three sites within the capital city of Austria [76].

#### 2.1.2. Czechia

Based on older data from the literature, the Czech Republic is regarded to be an endemic country of both *D. immitis* and *D. repens* species. However, while cases of *D. repens* are commonly reported in dogs in South Moravia, no recent reports of *D. immitis* are available. In the Czech Republic, *D. immitis* was only reported by Svobodová et al. [77,78]. This steeply contrasts the virtual absence of heartworm disease in the Czech Republic, and the autochthonous infection by the parasite has not been detected since then. Two recent studies [79] failed proving *D. immitis* in large sets of dogs examined by rapid diagnostic devices and PCR. Imported cases are sporadic and usually associated with imports of dogs from endemic regions in South Europe. Cases are not systematically reported by private vets.

*Dirofilaria repens* is well-known among veterinary clinicians in South Moravia, and its presence was also confirmed in mosquitoes [80]. Two studies reported human cases in the same region [81,82]. In the most recently published study, Miterpáková et al. [79] provided comparative data on distribution of *D. repens* in Slovakia and the Czech Republic, reporting 1.9% prevalence of *D. repens* in dogs from the Czech Republic. However, distribution shows a strong geographic pattern and is not homogenous; prevalence in endemic regions is higher. *Dirofilaria repens* is well-established in the domestic dog population in lowland areas along the Dyje and Morava rivers, extending northwards to the Kroměříž region. This distribution corresponds well with previously diagnosed cases of human subcutaneous dirofilariosis [81,82].

#### 2.1.3. Germany

In Germany, reports on imported *Dirofilaria* spp. infections started at the end of the last century, although no epidemiological framework was usually given. For the period of 1991–1993, Leuterer and Gothe [83] identified *D. repens* and *D. immitis* infections in three and 12 dogs, respectively, which had been imported from or traveled to endemic areas. In 1993–1996, a total of 155 imported or traveled dogs were diagnosed with filarioid infections [84,85,86,87], 10 of them with *D. repens*, 115 with *D. immitis*, and one dog with a coinfection of *D. immitis* and *Dipetalonema reconditum*. Two other dogs were mono-infected with *D. reconditum*, whereas for the remaining 27 Knott´s test-positive dogs, native blood smears were not available for histochemical filariae identification.

In the following years, reports on the occurrence of dirofilariae in Germany stopped until the first autochthonous case of *D. repens* infection was diagnosed in a Southwestern German dog in 2004 [88]. Three years later, further autochthonous *D. repens* infections were reported from a sledge-dog kennel in Northeastern Germany [89], and the parasite was also identified in three of 44 Southwestern German hunting dogs with no history of traveling [90]. With these reports, prevalence data on *Dirofilaria* spp. in Germany gained importance, and retrospective studies including several thousand samples detected 1.1–3.1% *D. immitis* antigen-positive imported or traveling dogs during 2004–2008 [91,92,93,94]. In a similar period (2004–2009), Knott´s test revealed microfilariae in 6.4–7.7% of dogs; however, no species differentiation was carried out [93,94,95]. In a study only including traveling dogs, none of the individuals tested positive for *D. immitis* antigen (380 dogs) or microfilariae (223 dogs) [96].

Additionally, autochthonous *D. repens* infections stimulated research on mosquito vectors. While no *Dirofilaria* DNA was found in more than 80,000 mosquitos collected throughout Germany between 2009 and 2010 [97], *D. repens* DNA was observed in 2011 in a pool consisting of *Culiseta annulata*; in 2012, in two pools of *An. maculipennis* s.l. and each one of *Anopheles daciae* and *Aedes vexans*; and in 2016, in one *Anopheles messeae* pool [98,99,100]. Furthermore, *D. immitis* DNA was amplified in two *Culex pipiens/torrentium* pools in 2012 [100]. Both *D. repens* and *D. immitis* were found in mosquitoes originating from Southwestern and Northeastern Germany, more precisely from the federal states of Baden-Wurttemberg, and Berlin and Brandenburg, from which the autochthonous *D. repens* infections in dogs were also reported. Moreover, both federal states were considered to be climatically suitable for dirofilarial development in the mosquito vector and classified as risk regions for stable endemicity [101,102,103]. However, DNA detection in the mosquitoes just proves that they had a blood meal on a (local) microfilaremic animal, but cannot be equated with established transmission cycles. Nevertheless, Sassnau et al. [104] reported that the number of *D. repens*–infected individuals in the sledge-dog kennel increased from five in 2007 to 11 in 2012. Likewise, in the German federal state of Saxony-Anhalt, which borders Brandenburg to the west, an autochthonous infection was diagnosed in a dog in 2010 [105]; in 2014, the first German autochthonous human case was reported [106]. However, the screening of 122 red-fox and 13 raccoon-dog (*Nyctereutes procyonoides*) lung samples from 2009 [107], and of 1023 dog-blood, 179 red-fox-blood, and 195 red-fox-spleen samples from 2013 and 2014 [108] did not provide evidence of endemic occurrence of *Dirofilaria* in Brandenburg.

The most recent data on filarial infections in Germany refer again to imported or traveling pets. In dogs imported between 2007 and 2015, a prevalence of 7.3% was found in the 178 tested individuals. Of the 13 positive dogs, eight were diagnosed with *D. immitis*, three with *D. repens*, one with *D. reconditum*, and no differentiation was performed in one dog [109]. In 133 German dogs traveling to endemic areas in 2007–2018, one dog (0.8%) became infected with *D. immitis* [110]. Fortunately, the first data on cats living in Germany have recently become available. Of the 618 cats subjected to *Dirofilaria* spp. PCR included in the feline travel profile, one (0.2%) tested positive, but no further species differentiation was conducted [111].

Overall, as the German climate allows for dirofilarial development in the mosquito vector [101,102,103], imported and traveling pets should be thoroughly monitored and, if positive, treated against dirofilariae to prevent the autochthonization of *D. immitis* and endemization of *D. repens* in Germany.

#### 2.1.4. Hungary

On the basis of human cases reported since 1879 without confirming the identification of worms, Kotlán [112], Szénási [113] and others suspected that *D. repens* had been present in Hungary since the end of the 19th century. Although human dirofilariosis is not a notifiable disease in the country, a few dozen ocular and subcutaneous dirofilarioses were reported during the last two decades [113,114,115].

The first autochthonous *D. repens* infections of dogs were described only at the end of the 1990s [116,117]. In nationwide epidemiological surveys [118,119,120], 11.1–19.6% of dogs and two cats were positive for *D. repens* microfilariae. Many infected dogs probably remain undetected due to the subclinical nature of the disease, and to the lack of rapid and reliable diagnostic tools. A significant cluster of microfilaremic dogs were found in the southern part of the country [120], where *D. repens* was the most frequent filarioid parasite in mosquito samples [121]. These veterinary reports confirmed that this nematode species is present in local dogs, representing a continuous risk of human infection in many regions of Hungary.

Heartworm infection was pathologically diagnosed only in dogs imported from the USA until 2000 [122,123]. The first autochthonous *D. immitis* infection was detected in a Hungarian Vizsla dog that lived in the eastern part of the country [124]. Since that time, the examinations of dogs [120,125,126,127,128], red foxes, golden jackals (*Canis aureus*) [129], and one ferret (*Mustela furo*) [130] revealed that *D. immitis* is endemic in the country, and the Great Hungarian Plain is hyperendemic. Mixed infections of dogs by both *Dirofilaria* spp. were also detected in some counties [120]. No human heartworm infection was diagnosed in Hungary.

It cannot be definitively excluded that *D. immitis* had been present in the country before the 21st century because no epidemiological surveys were carried out, and no reliable diagnostic methods were available earlier. However, it is more plausible that this filarioid species has only recently been introduced to Hungary, because neither its microfilariae nor adult worms were found in local dogs [118], and red foxes before [131]. Hunting dogs from Italy with patent heartworm infection may have acted as microfilarial reservoirs for the local mosquito population during their stay in the area, resulting in the development and transmission of infective L3 to native dogs. The role of infected wild canids arriving from neighboring countries might not necessarily be considered regarding the geographical distribution of heartworm infections in Hungary because only a few red foxes and two golden jackals were infected with a low number of worms without microfilaremia [129].

The occurrence and spread of both filarioid species in Hungary are not surprising, because the local climate and the abundance of mosquito vectors around their breeding sites offer suitable conditions for the development and transmission of these parasites. Stray dogs and dogs adopted from shelters pose an especially high risk in the epidemiology of both dirofilarioses because these animals are unlikely to receive proper examination and prevention.

#### 2.1.5. Luxembourg

So far, there are no data in the literature on infections with *D. immitis* and *D. repens* in Luxembourg. To the authors’ knowledge, no specific studies have been conducted on *D. repens* in Luxembourg. Between 2014 and 2020, the first serological tests were carried out, using the rapid diagnostic device to detect antibodies against *D. immitis*, among others. Serum from road-kill red foxes (n = 50) and raccoons (*Procyon lotor*) (n = 81) was analyzed across Luxembourg. All the tested red foxes and raccoons were negative. Given the presence of mosquitoes that can serve as vectors in Luxembourg (*Cx. pipiens* s.l. and *Ae. vexans* [132]), further studies should be conducted.

#### 2.1.6. Poland

The first autochthonous *D. repens* infections of dogs were reported in Central Poland, Mazovia between 2009 and 2011 [133,134,135,136]. Since then, the number of reported cases has been growing [137,138]. Epidemiological studies carried out in 2014 in the canine population in Mazovia revealed a surprisingly high prevalence of *D. repens*, especially in dogs from suburban and rural areas [139,140]. In a study of Demiaszkiewicz [139], 462 dogs aged 1.5–14 years were examined, using the Knott method. Microfilariae of *D. repens* were found in the blood of dogs originating from the city of Warsaw and from 18 districts of Mazovia (Mazowieckie). Overall prevalence was 25.8%. The highest prevalence (53.0%) and the highest intensity of infection were found in the Radom district (Southern Mazovia). In a study [140] among sled dogs living in Mazovia sampled between 2010 and 2013, *D. repens* DNA was detected in 15 of 34 dogs (44.0%). Prevalence was especially high (50.0–57.0%) in two sled-dog kennels situated near Grodzisk Mazowiecki (Southern Mazovia).

In a nationwide epidemiological study [141], 1588 dogs were examined for dirofilariosis. *Dirofilaria repens* microfilariae were found in 11.7% of the blood samples of dogs originating from all 16 provinces of Poland. The highest prevalence (25.8%) was found in Mazovia, Central Poland. About 12.0–16.0% of dogs were positive in Eastern Poland, while much lower prevalence was noted in western and northern areas of the country.

In another study [18], 147 blood samples from cats from Central Poland, and 257 blood samples from dogs from Central, Northern, Southern, and Western Poland were collected in the period of 2013–2015. No positive dogs were noted from Kraków (Southern Poland), Wrocław (Western Poland), and Gdańsk (Northern Poland). The DNA of *D. repens* and/or *Wolbachia* was identified in two cats (1.4%) from Central Poland. The DNA of *D. repens* was detected only in dogs in Mazovia (38.0%).

In the most recent studies, the prevalence of *D. repens* in dogs from the area of Poland was about 12.0% (2017, 2019, and 2020) [11,142]. This decrease in prevalence was accompanied by increased awareness of this parasitosis, both among dog owners and veterinary practitioners, and may reflect the increased application of preventive measures during the season of mosquitoes activity [11]. The DNA of *D. repens* was detected in samples containing a mixture of *Cx pipiens* and *Ae. Vexans* mosquitoes, collected in Mazovia during the summer months of 2010–2012 [143].

The awareness and endemic status of dirofilariasis due to *D. repens* have risen and been confirmed only in the last decade, following the recognition of autochthonous cases in dogs and humans. The first cases of human dirofilariasis (*D. repens*) preceded the reports on *D. repens* in dogs, and were between 2007 and 2009 [144,145,146]. In 2012, a paper reviewing the cases of dirofilariasis was published [147]. Between 2007 and 2011, a total of 18 *D. repens* infections were detected in humans in Poland. Parasitic lesions were located in various parts of the body in the form of subcutaneous nodules containing single nematodes surrounded by granulation tissue (15 cases). In three cases, subconjunctival localization was found. Of the 18 described cases, 17 were in Central Poland. In this area, autochthonous infections were identified in three women who had never left Poland. The first was found in 2010 in Grójec, and the next two in 2011 in Białobrzegi and Warsaw [147]. Since that time, the number of published human cases has increased, with reports on the unusual localization or manifestation of *D. repens* [148,149,150,151].

In 2012, the first, likely autochthonous, case of *D. immitis* infection was recognized in a dog in Gdynia, Northern Poland [152]. No additional cases have been reported to date, imported or autochthonous [142,148]. Results of three epidemiological studies revealed very low or zero prevalence: in the largest study in 2014, 3094 healthy dogs from the area of Poland were tested by rapid diagnostic devices. Only 0.2% of the samples tested positive (n = 5), with no information on clinical signs or origin (imported vs. autochthonous) of dogs [153].

In 2019, a rapid diagnostic device test was carried out on 167 healthy sled dogs from Lithuania (n = 46), Latvia (n = 24), Estonia (n = 20), and Poland (n = 35), and on 42 healthy pet dogs from Poland, including 20 dogs positive for *D. repens* [11]. No positive results were obtained, and no cross-reaction with *D. repens*–infected dogs was detected.

In 2020, 160 dogs from Eastern Poland were tested for *Dirofilaria* spp. (PCR, rapid diagnostic device). These dogs were selected on the basis of demographic features (kept outdoors, no ectoparasite prophylaxis) and the presence of clinical signs compatible with *D. immitis* infection (exercise intolerance, cough). Microfilariae of *D. repens* were identified by PCR in 20 dogs (prevalence, 12.0%), but no samples tested positive for *D. immitis* [142].

#### 2.1.7. Slovakia

In Slovakia, autochthonous canine dirofilariosis was recorded for the first time in 2005, when *D. repens* was confirmed in 13 dogs, and *D. immitis* in two other dogs from Bratislava and Komárno districts situated in the southwestern part of the country bordering Austria and Hungary. All infected dogs were asymptomatic [154].

In February 2007, the first monitoring covering two areas of Southwest and Southeast Slovakia was carried out. The study, encompassing 287 dogs of different age, gender, and breed, revealed microfilariae in 99 dogs, representing an overall prevalence of 34.5%. In all positive dogs, *D. repens* was detected, and in six of them, coinfection with *D. immitis* was confirmed. Only in seven dogs could an autochthonous source of the infection not be unambiguously evidenced, and only in four infected dogs was a health state alternation, including dermal changes, observed. Within this research, the utilization of dogs was revealed as an important risk factor for the infection. Police, guard, and hunting dogs, with prevalence rates of 51.1%, 50.0%, and 40.0%, respectively, were more often found to be infected when compared with companion dogs (an average prevalence rate of 7.8%). On the basis of this study, in the territory of Slovakia, highly endemic areas of *D. repens* were identified [155].

Between September 2007 and February 2010, a monitoring program of canine dirofilariosis aimed at working (police and military) dogs was performed in Slovakia. All 710 (591 police and 119 military) dogs from all Slovak regions were examined for *Dirofilaria* spp. presence. Microfilariae were detected in blood of 128 (18.0%) dogs (118 police and 10 military). DNA analyses revealed *D. repens* mono-infection in 125 dogs and mixed *D. repens*/*D. immitis* infection in three dogs. This survey confirmed the highest prevalence rates in southwestern parts of Slovakia identified as endemic for *D. repens* in previous study. In all infected dogs, the autochthonous origin of the infection was acknowledged. Evaluating the questionnaire data, it was highly presumable that the majority of the examined police dogs had become infected during their stay in training and breeding centers situated in the endemic area of Western Slovakia [156].

A comprehensive study summarizing research of canine dirofilariosis in Slovakia between 2005 and 2015 was published in 2016 [157]. During the 10-year study, a total of 4043 dogs from all Slovak regions were examined for *Dirofilaria* spp. Microfilariae were found in the peripheral-blood system of 450 dogs, representing an average prevalence of 11.1%. DNA analysis confirmed *D. repens* mono-infection in 440 animals, mixed *D. repens* and *D. immitis* infection in nine dogs, and one dog was infected only with *D. immitis*. The spatial distribution of *Dirofilaria* spp. showed significant regional differences. The highest above-average prevalence rates were steadily recorded in the southern regions of Nitra (over 25.0%), Trnava (18.4%), and Košice (12.7%). In the northern regions of Slovakia (Žilina and Prešov) bordering Poland, prevalence ranged between 2.0% and 4.0% [157].

An independent serological study tested newly developed commercial rapid diagnostic devices in 2015. During this study, blood and sera from 180 dogs originating from the southwestern and southeastern regions of Slovakia were investigated for the presence of microfilariae and circulating *D. immitis* antigen. Microfilariae were observed in 12 of 180 examined dogs, and subsequent DNA analyses confirmed *D. repens* in all the positive samples. In parallel, using the rapid diagnostic device, circulating *D. immitis* antigens were detected in the serum samples of five dogs (2.8%). In two *D. immitis*-seropositive dogs, microfilariae of *D. repens* were also found. Regarding DNA analyses not revealing *D. immitis* presence, all five cases can be considered to be an occult form of the infection. One of the *D. immitis*–positive dogs came from Southeast Slovakia, and the remaining four from Komárno district, in the southwest, where *D. immitis* was confirmed in previous studies [158].

However, after 2015, an evident increasing trend of *D. immitis* cases in Slovakia has been observed. The first outbreak of heartworm infection was recorded in a dog-breeding establishment in the district of Dunajská Streda, Trnava region, near the border with Hungary. Out of 25 examined dogs (22 Newfoundlands, two Central Asia shepherd dogs, and one Sarplaninac), dirofilariosis was diagnosed in 18 animals (72.0%), using several different diagnostic approaches (Knott test, DNA analysis, histochemical staining, rapid diagnostic device). Ten of the infected dogs were positive only for *D. immitis*, two for *D. repens*, and mixed infection was confirmed in six dogs. Occult *D. immitis* infections without circulating microfilariae were recorded in six dogs. No dogs showed clinical signs of heartworm disease. Regarding travel history, the autochthonous origin of the infection could unambiguously be confirmed in seven dogs [159].

The first registered fatal case of canine heartworm disease was recorded in 2019. In two seven-year-old Tibetan Mastiff siblings from the Košice region, Southeast Slovakia, raised in the same household, *D. immitis* was confirmed. The course of the infection in the two dogs markedly differed. Although the female dog manifested no health-status alternation, the male dog exhibited severe clinical signs, including elevated creatinine and urea levels, increased liver hyperechogenicity, and hepatomegaly. The dog died five days after hospitalization. Subsequent postmortem examination revealed adult *D. immitis* worms in the right heart ventricle [160].

The most recent epidemiological study on canine dirofilariosis in Slovakia was carried out in late 2019. Within the study, 644 randomly selected dogs were examined for the presence of *Dirofilaria* spp. Microfilariae were present in 68 blood samples with an overall prevalence of 10.6%. Subsequent DNA analysis confirmed *D. repens* mono-infection in 38 (5.9%) dogs, a single *D. immitis* infection in 21 (3.3%) animals, and both *Dirofilaria* species were detected in nine (1.4%) samples. These data indicate an increasing number of *D. immitis* cases in Slovakia, previously considered to be endemic only for *D. repens* [79].

In Slovakia, besides dogs, the presence of *D. repens* DNA was confirmed in the spleen samples from one individual of beech marten (*Martes foina*) and red foxes [161,162]. The results of the study showed 105 of the 183 examined red foxes being infected, representing an overall prevalence above 57.0%.

Regarding *Dirofilaria* vectors, research focused on mosquitoes is still in its infancy and mostly regionally oriented in Slovakia. The first screening for dirofilariosis in mosquitoes was performed in 2013 in Eastern Slovakia, and showed that the *Ae. vexans* species was incorporated into the life cycles of both *D. repens* and *D. immitis* [163,164]. During the next xenomonitoring carried out in Bratislava, Western Slovakia, *D. repens* was detected in *An. Messeae*, *An. maculipennis*, and *Cx pipiens* complexes, and *D. immitis* in *Coquillettidia richiardii* and *Cx. pipiens pipiens*. Both dirofilarial species were also found in *Ochlerotatus sticticus* [165].

The first case of human dirofilariosis in Slovakia (at that time Czechoslovakia) was reported in 1992, when the presence of a wormlike formation in the vitreous body of a patient was discovered at the ophthalmological examination. Nevertheless, retrospective view of this case reveals some doubts about the diagnosis [166]. The first autochthonous and unambiguously confirmed case of human dirofilariosis was reported in 2007, two years after the first finding of dirofilarial parasites in dog population. Since then, between 2007 and 2020, 23 cases (subcutaneous, ocular, and pulmonary) were confirmed in Slovakia. In all cases, *D. repens* was validated as the causative agent [167,168].

#### 2.1.8. Slovenia

The first case of *D. immitis* in Slovenia was recorded in 1986. A clump of nematodes was found in the right ventricle and pulmonary artery of only one dog. The researchers hypothesized that a factor in the spread of dirofilariosis in Slovenia might be dogs imported from Italy and tourism flows with pets, which led them to expect an increase in the number of infected dogs. They cautioned that, with these epidemiological data, human cases should also be expected. They assumed that, since *D. repens* was present in neighboring Italy, its occurrence in Slovenia should also be expected [169]. The following year, the first case of the subcutaneous form of dirofilariosis in a red fox, caused by *D. repens*, was described in Slovenia by Brglez and Verbančič [170]. They found a high number of mature and juvenile parasites in a red fox killed on the road. In 1998, a human case of subcutaneous dirofilariasis was described in the occipital region of a 61-year-old Slovenian woman, caused by *D. repens*. The authors identified a trip to Canary Islands, Spain as the probable site of infection, because the subcutaneous tumor was diagnosed seven months later. The authors suggested that human cases of dirofilariasis are most likely under-reported, as many cases are undiagnosed or unpublished [171]. Currently, there is no officially reported number of human cases of dirofilariasis in Slovenia. A study of imported canine filarioid infections in Germany from 2008 to 2010 reported that *D. repens* was found in a dog imported from Slovenia. Although no prevalence studies of dirofilariosis in dogs were available, the authors considered Slovenia to be endemic for *D. repens* [92]. Currently available data from the Institute of Microbiology and Parasitology at the Veterinary Faculty of the University of Ljubljana show that, out of 400 blood samples from dogs acquired and tested for *Dirofilaria* spp. between April and October 2018, only two were positive for this parasite (Vergles Rataj, personal communication).

#### 2.1.9. Switzerland

Due to the perceived spread of *D. immitis* in the USA in the 1960s [172], the presence of the parasite in countries neighboring Switzerland, such as Italy [173,174] and France [175,176], and the report of an imported case in Germany [177], in a review article, Thun (1975) alerted Swiss veterinarians about the relevant aspects of *D. immitis* infections in dogs [178]. At the end of the 1980s, the first imported cases of canine dirofilariosis were diagnosed at the Institute of Parasitology, University of Zurich by the detection of circulating antigens and characterising microfilariae by acid phosphatase activity. The first clinical case of *D. immitis* dirofilariosis was diagnosed at the Animal Hospital of the Veterinary Faculty in Zurich, in a Siberian husky living in Milan (Italy) [179]. While clarifying the situation of another husky living in the same kennel, this dog was negative for *D. immitis* but positive for microfilariae of *D. repens*, thereby representing the first diagnosed and imported case of cutaneous dirofilariosis in a dog in Switzerland. Moreover, two stray dogs of unknown origin were diagnosed positive for *D. immitis* in Ticino, Southern Switzerland, and several dogs originating from the Mediterranean basin were diagnosed positive for *D. immitis* or *D. repens* at the Institute of Parasitology in Zurich [179]. In a follow-up study in which 217 stray dogs and 154 unwanted dogs from Ticino had been investigated, microfilaria were isolated from the blood of four dogs; these were confirmed as *D. immitis* by morphology and antigen detection. In all these cases, the import of dogs from Italy could not be excluded, thus not confirming the autochthonous presence of *Dirofilaria* spp. in the country. However, on the basis of further mentioned cases diagnosed close to the border with Italy and in suitable temperatures for the development of *D. immitis* in mosquitoes in the same area, the establishment of the parasite was anticipated [180]. In fact, when testing 479 blood samples from that region, three (0.6%) and eight (1.6%) dogs were positive for *D. repens* and *D. immitis*, respectively. For a single dog, local transmission was confirmed by excluding traveling abroad by the owner [181]. The investigation of dog samples from both sides of the borders, Switzerland and Italy, confirmed higher prevalence in Italy, while contemporaneously identifying four dogs positive for *D. repens* (n = 2) and/or *D. immitis* (n = 3) from Ticino. Due to the limited number of cases in Southern Switzerland despite the widespread presence of suitable vectors [182,183], Southern Switzerland is considered as the border of the endemic area of both *Dirofilaria* spp.; therefore, prevention measures were recommended and are currently regularly implemented. These include the treatment of all infected dogs with microfilariae in order to decrease the risk of transmission [184]. To our knowledge, no autochthonous case of dirofilariosis north of the Alps was determined (Deplazes, personal communication). In fact, most of the cases have a clear history of import [185] or traveling.

Similarly, three human patients of dirofilariasis diagnosed in Swiss hospitals originated from abroad (India) [186], had a travel history to the Mediterranean area [187] or to Southern Switzerland and Northern Italy [188]. Different organs were affected: the epididymis in the first case, pulmonary nodules in the second, and the subconjuctival tissue in the third patient. Two were confirmed as caused by *D. repens*, and one was attributed to *D. immitis* (pulmonary nodules), but not confirmed by laboratory techniques.

### 2.2. Northern Europe

Results of a questionnaire study among veterinarians showed that 11.0% of the participating veterinarians who were practicing in Nordic countries reported having seen dogs with *D. immitis* infection, 3.0% reported having seen dogs with *D. repens* infection in 2016, and a majority of the cases were reported to be in dogs with a history of travel or import [56]. The situation was very different to that in the nearby Baltic countries, in particular regarding *D. repens*: almost a fourth of veterinarians practicing in Baltic countries reported having seen dog(s) with *D. repens* infection, and none of these had a history of travel or import [56]. *Dirofilaria repens* emerged in the Baltic countries in 2008–2012 and became endemic [11,13,189,190].

#### 2.2.1. Denmark

There are no published reports of *Dirofilaria* spp. in animals in Denmark, while a human case of *D. repens* in a 39-year-old woman was reported in 2014. However, the woman was likely infected on Crete [191]. The vectors of both *D. repens* and *D. immitis* are present in Denmark (Huus Petersen, personal communication) and, during a period of 15 years, two locations in Denmark reached in July at least once the 130 heartworm development units (HDU, the total environment heat required for the development of *Dirofilaria* from microfilaria to infectious L3 within the mosquito), but none of them reached the 130 HDU based on average temperature [192]. There are no published records of surveillance studies on dirofilariosis in wild-living canines from Denmark, and to the authors’ knowledge, no surveillance studies have been performed.

#### 2.2.2. Finland

There is one published case report of autochthonous human *D. repens* infection from Southeast Finland from 2015 [193]. There are no published reports of autochthonous *D. repens* nor *D. immitis* infections in dogs from Finland. The first *D. repens* finding in an imported dog was in 2014 [193]. *Dirofilaria ursi* is present in brown bears (*Ursus arctos*) in the eastern part of Finland, but no human cases have been reported [193,194]. During a period of 15 years several places in Finland reached in July at least once the 130 HDU, but none of them reached the 130 HDU based on average temperature [192].

#### 2.2.3. Iceland

There are no reported cases of *D. immitis* or *D. repens* from Iceland. Moreover, competent mosquito vectors are not present on Iceland.

#### 2.2.4. Norway

The first published case report, published in 1991, of apparently imported human *D. repens* infection in the Nordic countries was from Norway [195]. *Dirofilaria repens* infection was reported in dogs imported to Norway from South Africa [196] and Hungary [197]. *Dirofilaria immitis* was also reported in imported dogs [198]. During a period of 15 years, some places in Southern Norway reached in July at least once the 130 HDU, but none of them reached the 130 HDU based on average temperature [192].

#### 2.2.5. Sweden

Endemic cases of *Dirofilaria* spp. have not been found in Sweden to date, but in addition to *A. vasorum*, these infections are notifiable, and the following cases were reported during the last five years: three cases in 2015, two cases in 2016, no cases in 2017, two cases in 2018, and seven cases in 2019 [199,200]. Unfortunately, *Dirofilaria* spp. infection is reported at the genus level, i.e., the record of notified cases is not discriminating between *D. immitis* and *D. repens*, so it is not possible to describe which of the two parasites has been diagnosed more often. According to the most recent studies of the northward expansion of *Dirofilaria* infection in Europe, during a period of 15 years some places in Sweden reached in July at least once the 130 HDU, but none of them reached the 130 HDU based on average temperature [192].

## 3. *Angiostrongylus vasorum*

### 3.1. Central Europe

#### 3.1.1. Austria

*A. vasorum* was detected in gastropods in Austria in two studies [51,201]. One study investigated their occurrence in 1320 gastropods collected in the Austrian provinces of Styria, Burgenland, Lower Austria, and in metropolitan Vienna. Metastrongyloid larvae were microscopically detected in 25 samples, and sequence analysis confirmed the presence of *A. vasorum* in one slug (*Arion vulgaris*; 0.1%) [51]. The first cases of canine angiostrongylosis reported in Austria were imported from endemic areas of France [202,203]. In a retrospective study, 1040 fecal samples of Austrian dogs were analyzed by using the Baerman method [204]. L1 of *A. vasorum* were documented in 1.3% of the dogs originating from Vorarlberg (Western Austria), Styria (Southeastern Austria), Lower Austria, and Vienna (Northeastern Austria). Moreover, 1.2% of 1279 dogs were positive for specific antigens, and 1.5% for specific antibodies at serological tests. These dogs originated from all Austrian provinces (with the exception of Burgenland), namely Lower Austria, Upper Austria, Vienna, Styria, Carinthia, Salzburg, and Tyrol [204]. However, although helminth L1 antigens and antibodies were reported at many locations, these data indicate a very low prevalence of *A. vasorum* in dogs in Austria [204].

#### 3.1.2. Czechia

The occurrence of *A. vasorum* was not surveyed on larger sample set. A relatively recent study by Hajnalová et al. [205] found 4.7% of dogs (nine of 193) to be positive for circulating antigen by ELISA. L1 were detected in one of the 253 examined dogs. Infection is sporadically detected in necropsied red foxes; however, systematic research is not conducted. On the basis of feedback from small-animal practitioners, *A. vasorum* is not yet considered an issue, though conditions for the transmission are ubiquitous.

#### 3.1.3. Germany

The first reference on angiostrongylosis in Germany describes phagocytosis of the parasite by giant cells in a histological section of a five-year-old royal poodle suffering from verminous pneumonia caused by *A. vasorum*, necropsied in 1964 [206]. Shortly thereafter, angiostrongylosis was reported in a four-year-old dachshund that was euthanized in 1965, due to incurable heart damage [207]. In both cases, nothing is mentioned about a possible travel history, so it remains unclear whether the dogs had traveled or not. A few decades later, in 2003, another case was diagnosed in a Southern German dog living on the border to Switzerland. On the basis of anamnestic data, the authors considered an autochthonous infection [208], whereas subsequently published cases reported an import from or travel history to France, Italy, or Portugal [209,210].

The first German prevalence data are available from 1999 to 2002, where *A. vasorum* L1 were found in 0.3% of diagnostic dog fecal samples [211]. In the following years, prevalence increased to 0.9% in 2010 and 1.6% in 2016 [212,213]. *A. vasorum* occurrence spatially clustered in Southwest Germany. This pattern was also observed in a seroprevalence study resulting in 0.5% antigen-positive and 2.3% antibody-positive dogs; however, the vast majority of samples originated from West German federal states [214]. In a study conducted in Central Germany, 1.2% clinically healthy sheep-herding dogs tested coproscopically positive [215].

In dogs with clinical signs indicative for lungworm infection, *A. vasorum*-positive fecal samples ranged between 1.1% and 7.4% [216,217,218,219]. The most recent study on lungworm-suspected dogs evaluating more than 12,000 fecal samples reported both a significant increase in *A. vasorum* prevalence, and an accumulation of positive dogs in Northeast and Southwest Germany, indicating a potential spread or awareness of the parasite (from these parts of Germany, autochthonous *D. repens* infections were also reported; see above section). A study in red foxes confirmed the endemicity of *A. vasorum* in Northeast Germany by DNA detection in 9.0% of the lungs [107]. Nevertheless, the prevalence of 27.0% in red foxes in Rhineland-Palatinate [220] still reveals Southwest Germany to be a highly endemic region [221].

Lastly, the increasing *A. vasorum* prevalence and the accumulation cluster in Southwest and Northeast Germany raise the question of what happens in the intermediate parts of the country. New data are desirable, especially as samples from these intermediate regions were underrepresented in all studies evaluating geographical distribution.

#### 3.1.4. Hungary

In 1960, Kotlán [222] first mentioned the sporadic occurrence of *A. vasorum* in a dog and red foxes in Hungary. A few decades later, angiostrongylosis was found in red foxes [129,131] and golden jackals [49], indicating that wild canids play an important role in the distribution and establishment of this parasitic species, since the mollusc intermediate hosts are broadly distributed in Hungary [223]. The first cases of dogs infected by *A. vasorum* were two asymptomatic animals kept in gardens close to the Croatian border, where five slugs were found carrying larvae of this parasitic species [224]. These infections were considered autochthonous because both dogs were born where they lived and never left their villages. In a large-scale combined serological survey of 1247 pet dogs, 1.4% of them were positive by two ELISA [225]. A considerable number of cases were observed in Budapest, and in the southern part bordering Croatia. The results of this serological survey confirmed the endemic occurrence of *A. vasorum* in dogs in different parts of Hungary.

#### 3.1.5. Luxembourg

There are no reports in the literature on the presence of *A. vasorum* in Luxembourg. In a current study (Heddergott, personal communication), 27 fresh road-kill red foxes, mainly from the eastern part of the country (administrative districts Diekirch, Echternach, and Grevenmacher), were examined for infection with *A. vasorum*. At necropsy, the heart, lungs, and adjacent vessels and from the rectum of the cadavers were taken. The genetic diagnosis of fecal samples was performed by SAF technique, *Giardia* and *Cryptosporidium* coproantigen ELISAs, and by duplex copro-PCR. All examined red foxes were negative.

#### 3.1.6. Poland

The first finding of *A. vasorum* in Poland (northeast, Augustowska Primeval Forest) was in red foxes in 2013 [226]. Adult nematodes were found in 4/76 red foxes (5.0%). In 2014, the first clinical case was described in a dog in Lublin (Eastern Poland) [227].

In a large epidemiological study conducted in 2013, the sera of 3345 healthy dogs from veterinary clinics all over the country were tested; specific antibodies against *A. vasorum* were found in 60 animals (1.8%), and parasitic antigens in 43 dogs (1.3%) [228].

In another study, *A. vasorum* L1 were detected by using coproscopic methods in 7.0% of the 58 fecal samples of grey wolves from the Bieszczady Mountains (Southeast Poland) [229].

#### 3.1.7. Slovakia

The first autochthonous case of canine angiostrongylosis in Slovakia was officially reported in 2013 in a seven-month-old Maltese pinch dog in Košice, southeastern part of the country [230]. The physical examination revealed no remarkable clinical signs in the patient [230].

In the same year and location, *A. vasorum* was diagnosed in an 18-month-old Bernese mountain dog. The infection was accompanied by serious clinical signs and almost fatal course; inter alia, irritating cough, dyspnea, vomiting, bilateral scleral bleeding, and acute physical collapse were observed in the patient. The infected dog excreted L1 in high numbers (more than 800 L1 were counted in 10 g of feces) [231].

On the basis of these first cases, a serological survey was conducted to assess the current distribution of *A. vasorum* in the dog population of Slovakia. Serum samples from 225 dogs originated from 22 districts were tested by ELISA for the presence of circulating *A. vasorum* antigens and for the detection of specific antibodies. Fourteen (6.2%) dogs were positive in at least one ELISA; seven dogs (3.1%) were only antibody-positive, four animals (1.8%) were positive only for circulating *A. vasorum* antigen, and three individuals (1.3%) were positive in both ELISAs. Seropositive dogs came from different regions with the highest accumulation of the cases in Southwest Slovakia. Three dogs positive for circulating antigen and specific antibodies originated from Bratislava region on the border with Austria [232].

Another survey based on the Baermann technique and modified-flotation method revealed that 14 of 339 (4.1%) examined dogs had been infected by *A. vasorum* [233].

A rare case of canine angiostrongylosis was described in 2019, when *A. vasorum* was detected in the anterior eye chamber of an 18-month-old beagle from the northeastern part of Slovakia. The dog’s feces were examined for the presence of L1 with negative results, but the final diagnosis was confirmed by DNA analyses and sequencing [234].

The circulation of *A. vasorum* in populations of free-living carnivores was confirmed in two independent surveys. Between 2014 and 2016, 571 fecal samples from red foxes, originating from all Slovak regions, were investigated for L1 of *A. vasorum*. The parasitic presence was confirmed in 31 animals, representing an average prevalence of 4.4%. In five positive red foxes, infection with *Crenosoma vulpis* was also diagnosed. Within this study, the potential influence of selected environmental variables on the occurrence of *A. vasorum* was quantified, using logistic regression. The distribution of *A. vasorum* showed typical spatial clustering and occurred in endemic foci mainly in the eastern part of Slovakia. A cluster of *A. vasorum* infection foci was found in both humid and the driest areas of Slovakia. A multivariable model for *A. vasorum* also revealed tendency of the parasite to prefer areas with higher shares of arable land and lower proportions of forests [48].

Besides the red fox, the grey wolf (*Canis lupus*) is considered to be another suitable reservoir host for *A. vasorum*. Between 2015 and 2016, the first systematic parasitological examination of the wolf population living in two national parks and in one protected landscape area of Slovakia was carried out. Overall, 256 wolf fecal samples were gathered and examined for *A. vasorum* presence, using the modified-flotation method with zinc sulfate solution. *Angiostrongylus vasorum* L1, subsequently confirmed by DNA analysis, were detected in two samples, in one wolf originated from Tatra National Park and in one individual from Poľana Protected Landscape Area [235].

#### 3.1.8. Slovenia

In several dozen samples per year sent to the Institute of Microbiology and Parasitology at the Veterinary Faculty of the University of Ljubljana for diagnosis of lungworms, *A. vasorum* was diagnosed only once in a hunting dog imported from Hungary, whose cause of death was angiostrongylosis (unpublished data).

#### 3.1.9. Switzerland

In Switzerland, the first cases of *A. vasorum* were reported from a dog-breeding station in Zurich in 1968 [236], but only decades later did Staebler et al. [208] report five dogs infected with *A. vasorum* in the northern part of Switzerland, and three dogs coming from Southern Ticino, all diagnosed between 1999 and 2004. In addition, in 2001, two infected red foxes originating from the region of Basel were reported [237].

By means of serological tests that were developed for circulating antigen and specific antibody detection [238], in a first epidemiological study with more than 4000 sera an overall seroprevalence of 1.0%, 2.8%, and 3.1% for dogs positive in both ELISAs, in antigen ELISA and antibody ELISA, respectively, was detected. Spatial analysis showed that positive dogs were distributed over large areas of the country, and a cluster of antibody-positive dogs in the northern area of Switzerland bordering Germany was identified [239]. Approximately in the same period, a grid-cell-based noninvasive fecal-sampling scheme for red fox samples indicated an overall prevalence of 8.8%, and revealed that land use and altitude affected prevalence rate [240]. Both dog and red-fox studies showed that prevalence rates increased with decreasing altitudes (and corresponding temperature variations), and that trend prevalence was higher in and around the first known endemic foci where *A. vasorum* was initially present. Investigating the Swiss red-fox population further, it was hypothesized that the transmission of *A. vasorum* among red foxes started to increase at the end of the 20st century due to the higher density of red foxes [241], increasing contamination of the environment, thereby infecting intermediate hosts and dogs. In fact, working up blood samples back from the past three decades from throughout the country, a drastic *A. vasorum* emergence from 2.4% to 62.0% was identified, reaching currently regional prevalence of more than 80.0% [4]. In particular, around 2000, a marked increase in seropositive red foxes correlated with the first accumulations of cases of canine angiostrongylosis. Locally, prevalence based on red-fox necropsy increased fourfold in only six years [4]. A group of captive meerkats (*Suricata suricatta*), housed in such a locally known highly endemic area was *A. vasorum*-positive, with L1 excretion in seven of 17 animals. Their natural infection was supported by the identification of positive mollusc intermediate hosts in their immediate surroundings [37]. The very first global identification of a naturally occurring infection with *A. vasorum* in a cat may trace back to a highly endemic area; however, because cats do not become patent, such infections are highly challenging to diagnose intra vitam and are possibly underestimated [41]. Overall, these data evidenced the important role of red foxes as reservoir hosts, and also helped to understand the increasing number of dog cases along with significant prevalence in the red fox populations in other European countries in the last decade.

### 3.2. Northern Europe

#### 3.2.1. Denmark

In Denmark, the first case of *A. vasorum* was described in 1983 in a five-year-old Cairn terrier from North Zealand [242]. The dog was euthanized due to bronchitis; at necropsy, *A. vasorum* were observed in the arteria pulmonalis, and the smaller arteria and arterioles. L1 were also demonstrated in a fecal sample [242]. The dog had visited Southern France several times and probably acquired the infection there. The next case was observed in 1989, also in a dog from North Zealand that had also been visiting France (Huus Petersen, personal communication). In 1990/1991, clinical cases were diagnosed in a considerable number of Danish dogs, none of which had ever been outside Denmark, but all from North Zealand [243,244]. In the same period, 12 of 15 adult red foxes from North Zealand were found positive for L1 in the feces, and/or adult *A. vasorum* in the right atrium of the heart and the pulmonary arteries [245]. The parasite had not previously been detected in a1973 parasitological survey of 100 wild red foxes from Denmark [246]. Since then, North Zealand has been a hyperendemic focus of *A. vasorum* in red foxes and domestic dogs for decades [245,247], while the parasite was either absent or with low prevalence in red foxes in the remainder of Denmark (0.0–1.1%) [247,248,249]. The latest study of *A. vasorum* in Denmark was conducted in 2017/2018 on 1041 wild animals, including 367 red foxes [40]. The study showed that *A. vasorum* prevalence in red foxes originating from the remainder of Zealand (37.0%) was now similar to the prevalence in the hyperendemic North Zealand (37.5%). This indicates that the hyperendemic area expanded to include all of Zealand [40]. In Jutland, the prevalence of *A. vasorum* in red foxes was much lower (1.7–2.3%), but higher than what had previously been reported. This indicates that *A. vasorum* is spreading in the red-fox population both in New Zealand and in Jutland. In addition, raccoon dogs (15 of 476) and polecats (*Mustela putorius*) (7 of 14) constitute a reservoir for *A. vasorum* in Jutland with prevalence ranging from 2.1% to 3.6% and from 50.0% to 100.0%, respectively.

#### 3.2.2. Finland

*Angiostrongylus vasorum* appears to be multifocally present in Finland. Two autochthonous *A. vasorum* findings in dogs, from 2014 and 2017, have been described in detail [53]. There is also a single case report of imported *A. vasorum* infection in a domestic dog [250]. A questionnaire survey among veterinarians indicated that a limited number of more domestic dogs with the infection would have been seen in the country, including a third autochthonous case [53]. The parasite was described in red foxes in the 1960s [251], and in a single red fox in the southern part of the country more recently [252].

#### 3.2.3. Iceland

A dog, a Siberian husky, imported in December 2017 to Iceland from Switzerland, was reported to be positive for *A. vasorum* [253]. In this study, more than 5000 imported dogs had been examined since 1989, and only this single *A. vasorum* case was found.

#### 3.2.4. Norway

No autochthonous *A. vasorum* findings have been reported from domestic dogs from Norway. The parasite was detected in red foxes in the country for the first time in 2016, and further findings were reported from active surveillance [254,255]. In 2019, *A. vasorum* was detected also in Northern Norway [256].

#### 3.2.5. Sweden

The first endemic case of *A. vasorum* was described in Sweden in 2003, when a dog from the island of Sydkoster, province of Bohuslän, was euthanized, and the diagnosis was confirmed at necropsy [257]. The demonstration of the endemic presence of the parasite came from the finding of parasitic L1 in two fecal samples from red foxes from Sydkoster; later, *A. vasorum* adults and L1 were found at necropsy in a dead red fox coming from the same island [257]. During 2011–2015, the parasite was detected in 0.7% of fecal samples (n = 20 of 2882 samples) analyzed with the Baermann test at the National Veterinary Institute (SVA, Uppsala, Sweden); these findings came from different parts of the country [54]. During the same period, *A. vasorum* was found at necropsy in red foxes, representing an occurrence ranging between 0.3% and 1.4% of necropsied red foxes, but the necroscopic investigations were not aimed at detecting specifically *A. vasorum* [54]. Regarding other potential final hosts, *A. vasorum* was not found in grey wolves (n = 20) hunted in Sweden [258]. A large national seroprevalence study was performed on serum samples collected between 2013 and 2014, and it showed that 0.1% of dogs were positive in both parasite antigen tests and antibody tests [54]. Since the disease is notifiable in Sweden, the following cases were reported during the last five years: 11 cases in 2015, eight cases in 2016, no cases in 2017, two cases in 2018, and two cases in 2019 [199,200].

## 4. Factors Influencing the Prevalence and Establishment of *D. immitis* and *D. repens*

According to Simón et al. [15], the transmission of *D. immitis* and *D. repens* is limited by two main preconditions: (i) the presence of a mosquito species capable of transmitting the parasite and (ii) the presence of a minimal number of dogs infected with adult nematodes shedding microfilariae. The distribution of *D. immitis* and *D. repens* is further influenced by human behavior (e.g., the housing conditions and travel activity of dogs, and imports), and climatic conditions allowing the presence of competent mosquito vectors and larval development [15]. Although infections with *D. immitis* and *D. repens* were documented in various wild canids, wildlife seem to play a limited role in the spread of these pathogens.

Three factors majorly impacted the prevalence, distribution, and establishment of populations of *D. repens* and *D. immitis* in Central and Northern Europe:

### 4.1. Dogs Staying Outside Overnight

**Figure 2:**
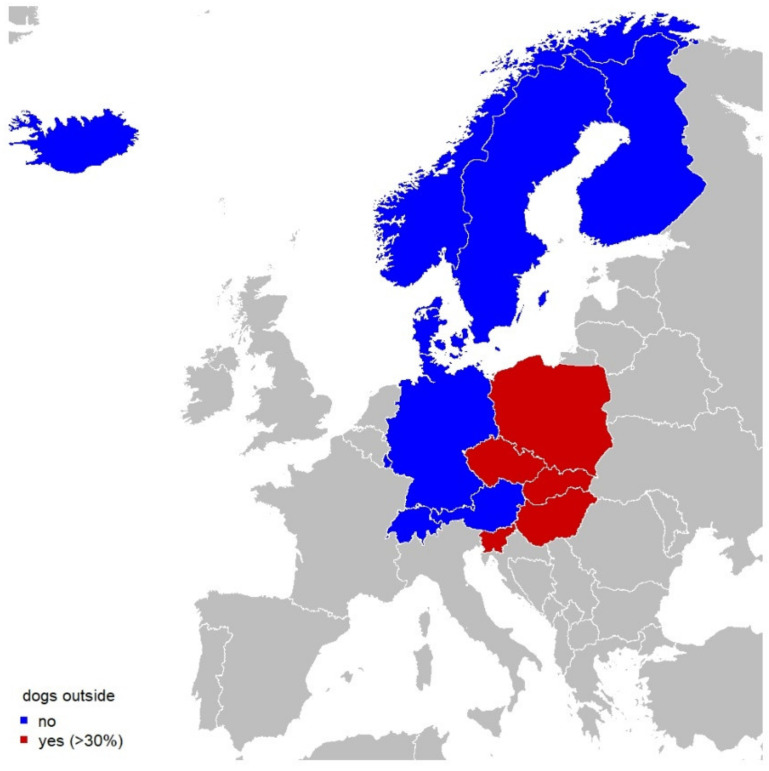
Dogs staying outside overnight (no = uncommon that dogs are kept outside overnight; yes ≥ 30% of dogs stay outside overnight).

Currently, human behavior is the major factor for the spread or import of *Dirofilaria* spp. in Central and Northern Europe. Both *D. immitis* and *D. repens* are frequently imported to non-endemic countries from endemic countries (e.g., stray dogs from Spain and Greece) [64]. The introduction of microfilaremic dogs to non-endemic areas may lead to local autochthonous outbreaks such as in military-dog facilities with kennel keeping [65]. The way of dog keeping majorly impacts the establishment of populations of these parasites. Stray dogs and cats, and private kennel keeping are not (very) common in Central and Northern Europe. Kennel or outdoor keeping of military, hunting, and sled dogs and keeping dogs in animal shelters are common practices in certain regions in Central and Northern Europe. In several Central European countries, more than 30.0% of the dogs are estimated to stay outside overnight (Figure 2), and so they are at a higher risk for mosquito bites compared to dogs staying inside. In addition, nocturnal house mosquitoes of the *Cx. pipiens* complex are competent vectors for *D. immitis* and *D. repens*, and those are the mosquitoes with the highest abundance in the vicinity of humans.

### 4.2. Diurnal Vector Activity

Diurnal vector activity: More than 60 mosquito species are vectors of *D. immitis* or *D. repens*. During blood meals, microfilaria are ingested, move to the Malpighian tubes, and L3 move to mouthparts and enter the labium (after passing the cibarium). Vector competence can be proven in laboratory studies [259]. At xenomonitoring studies where pooled mosquitoes are screened by PCR, vector competence can only be estimated [73]. Findings of L3 in caught wild mosquitoes (microscopically and not DNA in an entire mosquito only) can indicate vector competence. Furthermore, the molecular analysis of the head or thorax vs. abdomen can indicate vector competence [260,261]. However, laboratory suitability does not automatically mean that infections in the field occur frequently. Mosquito ecology and preferences differ from species to species (such as different habitats or blood-meal preferences) [262,263].

Several species of the *Aedes*, *Culex*, and *Anopheles* genera were demonstrated as competent vectors [15,262,264]. Synanthropic mosquito species with high abundance at human settlements might be the most important vectors for the establishment of parasite populations. In Central and Northern Europe, house mosquitoes of the *Cx. pipiens* complex fill this gap. These mosquitoes have a nocturnal activity pattern [265], so dogs staying outside overnight are more prone to these mosquitoes than those staying inside are.

**Figure 3:**
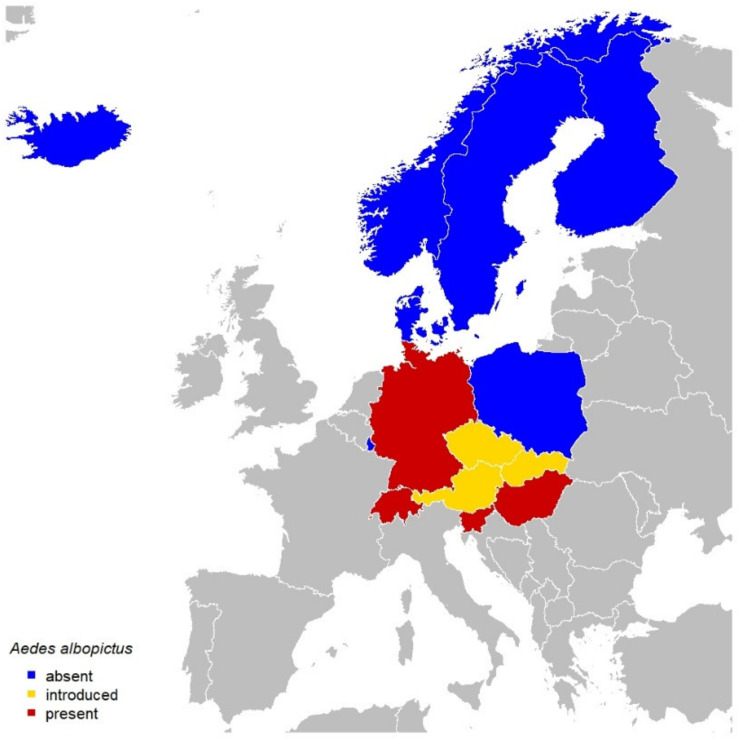
Distribution of Asian tiger mosquito (*Aedes albopictus*) in Central and Northern Europe according to the ECDC in March 2021 (introduced, no stable populations known yet; present, established populations at certain areas in the country). For detailed and updated information, please visit https://www.ecdc.europa.eu/en/disease-vectors/surveillance-and-disease-data/mosquito-maps (accessed on 14 August 2021).

In recent decades, several potential invasive mosquito species such as Asian tiger mosquito *Ae. albopictus* were introduced to Europe, primarily through the transport of goods (such as used tyres) [266]. In Southern Europe, the tiger mosquito rapidly established, and this invasive species is spreading northwards [267]. The tiger mosquito has already established in certain regions in Central Europe, and is regularly reintroduced in others (Figure 3) [75].

The Asian tiger mosquito is both a competent vector for *D. immitis* and *D. repens*, and an annoying day-active biter that outcompetes *Cx. pipiens* s.l. [268]. The establishment of populations of tiger mosquitoes would allow for the transmission of microfilariae to dogs during daytime and increase the risk of dogs to acquire an infection. The increased probability of *Dirofilaria* infections in areas where tiger mosquitoes established was reported from Italy [269,270].

### 4.3. Climate Change

Temperature has an important influence on the development of mosquito vectors and parasites. On the one hand, longer warm periods per year allow for more generations of parasites and vectors per year. On the other hand, increasing temperatures allow for a faster development from eggs to adult mosquitoes. Environmental temperature is also the key factor for microfilariae development in mosquito vectors (e.g., L3 require 16–20 days at 22 °C) [15]. Microfilariae do not develop to L3 at temperatures below 14 °C [27]. Several models showed that the expansion from Southern to Central and Northern Europe (but also in North America) is probable [27,192,271]. Both the heartworm predictive model (based on growing degree days) and the *Dirofilaria* development units show parts of Central Europe suitable for the establishment of these parasites [28]. In Central Europe, the possible transmission period for *D. immitis* is estimated to be three to four months, while 20 days to 2 months are estimated for some Northern European regions [15]. However, climatic changes might prolong these periods and allow for spreading to areas that are currently climatically unsuitable for these parasites and certain vectors.

## 5. Factors Influencing the Prevalence and Distribution Dynamics of *Angiostrongylus vasorum*

*Angiostrongylus vasorum* generally has patchy distribution with hyperendemic foci surrounded by low prevalence areas [272,273,274]. Its recent spread in various European territories calls for higher awareness by local veterinarians, as the absence of records in a given area may often be due to lack of information [8].

The emergence of *A. vasorum* in Central and Northern Europe is likely driven by a combination of factors, including wildlife movement to urban areas, increased dog movements, and possibly climatic changes.

Compared to *D. immitis* and *D. repens*, wildlife reservoirs are highly relevant for the distribution of *A. vasorum*. The prevalence of *A. vasorum* in wild canids in Europe, mainly red foxes and golden jackals, which act as natural reservoir of this parasite [4], is regionally high. Red foxes are ubiquitous, share recreational areas with dogs around urban contexts, and are usually subjected to reinfections that can lead to high worm burdens, as they do not reach effective immunity following *A. vasorum* infection [4,241,275]. As a consequence, they can act as a continuous source of environmental contamination, favoring the infection of gastropod intermediate hosts [276], and thereby the local establishment of the parasite [277].

Climatic changes may also play a role in the increase in the distribution of angiostrongylids, including *A. vasorum*, as their development from L1 to L3 inside snails may be positively influenced by the increase in environmental temperature, contrarily to crenosomatids [278,279,280,281]. Thus, it cannot be excluded that the current global warming may drive the further spread of *A. vasorum*. Water availability strongly affects the biology of intermediate *A. vasorum* hosts; thus, increased precipitations can act synergistically with increased temperatures in the spread of this metastrongyloid [274].

Lastly, dog relocation from endemic to non-endemic areas may cause the spread of parasites, including *A. vasorum*, and introduce the parasite into new areas [203,277,282,283,284,285,286,287].

Overall, the *A. vasorum* spread pattern seems to be multifocal and later coalescing, highlighting the need for larger and multicentral studies in order to support targeted interventions such as prophylactic anthelmintic treatments or testing [274].

## 6. Conclusions

*Dirofilaria immitis*, *D. repens*, and *A. vasorum* are spreading in Europe, and the relevance of these parasites is steadily increasing for dogs and veterinary practitioners in Central and Northern Europe. Housing conditions of dogs, increased animal movements, and climate change are important factors in the spread of these nematodes. Keeping dogs outside overnight seems to be a major factor for the establishment of *D. immitis* and *D. repens.* However, the establishment of invasive, diurnal, synanthropic, competent mosquito vectors such as *Ae. albopictus* may also influence the spread of these filarioid helminths. Although the reasons for the spread of *A. vasorum* are not definitely clarified, habitat sharing and increased chances of contact with red foxes seem to play a major role in the epidemiology of this parasite, which may also be influenced by increased temperature and precipitation, and dog relocations.

Research efforts focusing on these parasites vary by country, and cross-border studies are few. The available data are not easily comparable. Both *Dirofilaria* spp. and *A. vasorum* merit monitoring and further studies in Europe.

## Data Availability

No new data were generated for this review article.
